# Subacute Thyroiditis Masquerading as Fever of Unknown Origin

**DOI:** 10.7759/cureus.56576

**Published:** 2024-03-20

**Authors:** Stefan Gafoor, Raheem Robertson, Fawwad A Ansari, Sahar Karim, Zola Nlandu

**Affiliations:** 1 Graduate Medical Education, Piedmont Athens Regional Medical Center, Athens, USA; 2 Infectious Diseases, Piedmont Athens Regional Medical Center, Athens, USA

**Keywords:** acute suppurative thyroiditis, nonsteroidal anti-inflammatory drugs (nsaids), differential diagnoses, fever of uknown origin, subacute thyroiditis

## Abstract

Subacute thyroiditis (SAT) is an inflammatory disease of the thyroid gland. It can present with overlapping features of other etiology of thyroiditis. It can present with thyroid enlargement and systemic symptoms such as fever as well as neck pain and may be confused with infectious thyroiditis. It can be difficult to diagnose and present as fever of unknown origin (FUO). A good history, physical examination, laboratory investigation, as well as imaging may aid in the correct diagnosis and prevent the inappropriate use of antibiotics. Treatment is usually with nonsteroidal anti-inflammatory drugs (NSAIDs) as well as corticosteroids. We herein present a case of SAT presenting as FUO. We highlighted the importance of proper clinical evaluation, the importance of thyroid imaging, and how to differentiate other forms of thyroiditis.

## Introduction

Fever of unknown origin (FUO) is commonly caused by infections, malignancy, or autoimmune diseases. Endocrine pathologies such as subacute thyroiditis (SAT) are an unusual but significant cause of FUO [1,2]. SAT, also known as de Quervain thyroiditis, stands out as an inflammatory variant usually associated with respiratory viral illnesses, contributing to its non-suppurative nature [1].

Despite its rarity, SAT holds clinical importance due to its prevalence as the primary cause of thyroid pain. The incidence of the disease is 12.1 per 100,000 and predominantly affects females, with the highest incidence observed in the 30-40-year age group [3]. This disorder presents a clinical challenge, particularly in cases where it deviates from the typical course, and it usually presents with painful and tender anterior neck swelling in 70-90% of cases [1]. The hallmark of SAT is the transient nature of thyroid dysfunction, initiating with hyperthyroidism that persists for several weeks, followed by a phase of euthyroidism, and eventually transitioning into a hypothyroid state. While the majority of patients experience a full recovery of thyroid function, approximately 15% may continue to manifest hypothyroidism [4].

In this case report, we present a unique case of SAT in a patient who presented as FUO. Notably, imaging studies raised concerns of suppurative thyroiditis, prompting further investigation. However, a meticulous history-taking and comprehensive workup led to the conclusive diagnosis of SAT, highlighting the importance of considering this uncommon variant in the differential diagnosis of fever presentations. This case emphasizes the need for a nuanced approach to SAT, acknowledging its mimics and diverse clinical manifestations.

## Case presentation

A 65-year-old male with a past medical history of hypertension and infective endocarditis requiring aortic valve replacement presented with persistent fevers going on for a month prior to his presentation. Due to persistent fever, he had sought medical attention at an urgent care facility on three separate occasions and had a respiratory viral panel done that was negative, and each time, he had been prescribed antibiotics for a presumed bacterial upper respiratory tract infection. He finally presented to our facility with the chief complaint of persistent fevers for four weeks. He also reported symptoms of upper respiratory tract infection including chill, runny nose, and cough recently. He also reported other sick family members in his household. His home medications were warfarin, lisinopril, and metoprolol. He had no known drug allergies and reported a 15-pack-year history of smoking, which he had quit 20 years ago.

On presentation, the patient had a blood pressure of 173/104 mmHg, a heart rate of 93 beats per minute, a temperature of 98.6°F, and an SPO2 of 98% on room air. On examination, he appeared ill-appearing. An ejection systolic murmur was auscultated over his aortic area. There were no tremors, lid retraction, or lid lag appreciated. On the initial workup, his complete blood count (CBC) and comprehensive metabolic panel (CMP) were within normal limits, his international normalized ratio (INR) was 2.6 (normal <1), his prothrombin time (PT) was 30.2 seconds (normal 10-12 seconds), and his inflammatory markers revealed an erythrocyte sedimentation rate (ESR) of 48 mm/hr (normal <20 mm/hr), a C-reactive protein (CRP) of 3.2 mg/dl (normal 0.8-1 mg/dl), and a lactic acid of 0.8 mmol/L (normal <2 mmol/L).

Given that the patient had a prosthetic valve in situ and persistent fevers, he was commenced on empiric vancomycin and cefepime for possible infective endocarditis. A screening transthoracic echocardiography did not reveal any valvular vegetations. Subsequently, transesophageal echocardiography was performed which also did not reveal any valvular vegetations, and his blood cultures remained negative. Antibiotics were therefore discontinued. The patient, however, continued to spike fevers. On further history, he disclosed a week's history of progressively worsening anterior neck pain and swelling with associated odynophagia. The pain was described as constant, diffuse, sharp in nature, and non-radiating.

**Figure 1:**
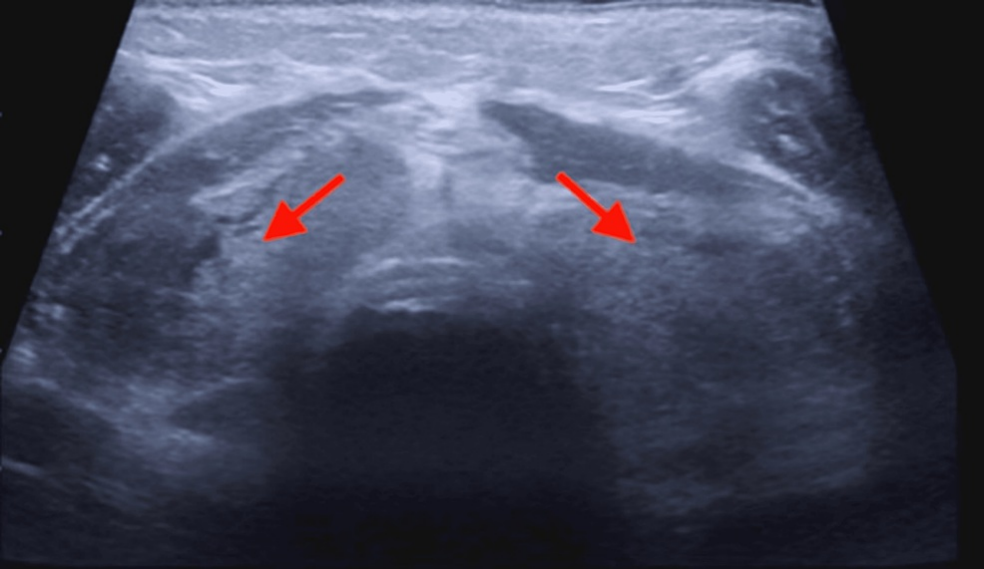
Ultrasound of the thyroid gland, demonstrating heterogeneous enlargement of both lobes of the thyroid gland (red arrows). No hypoechoic lesions or abscess was noted.

**Figure 2:**
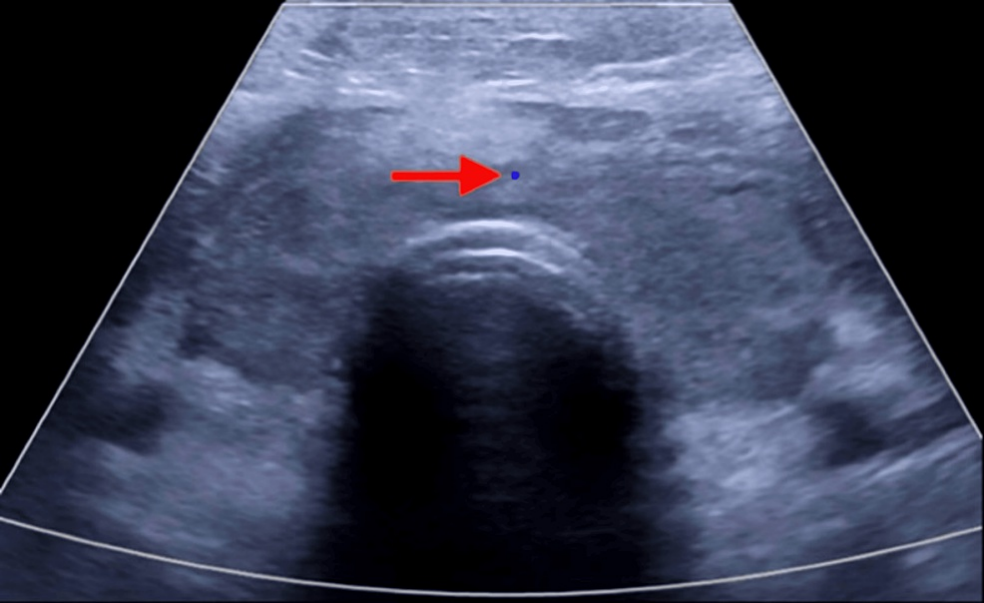
Color flow ultrasound of the thyroid gland, demonstrating a heterogeneously enlarged thyroid with reduced vascularity (red arrow).

A thyroid function test was obtained, and serum thyroid-stimulating hormone (TSH) was 0.4 uIU/mL (normal 0.4-5.5 uIU/ml), free thyroxine (T4) was 14 ng/dl (normal 0.7-2.1 ng/dl), and total triiodothyronine (T3) was 209 ng/dl (normal 60-200 ng/dl); with these findings, we considered the differential diagnosis of suppurative thyroiditis, SAT, and lastly autoimmune thyroiditis. We obtained a computed tomography (CT) scan of the soft tissues of his neck that showed an enlarged heterogeneous thyroid gland with fat stranding extending into the retropharyngeal and carotid spaces, features concerning suppurative thyroiditis. A subsequent thyroid ultrasound done revealed a heterogeneously enlarged thyroid without any hypoechoic lesions and no abscess. The vascularity was also decreased (Figure 1, Figure 2). Given these findings consistent with thyroiditis, a diagnosis of SAT was made; given that our patient was experiencing odynophagia, he was commenced on both intravenous methylprednisolone and ketorolac. His fever defervesced and other symptoms improved within 24 hours of commencing treatment. The patient was then discharged on day 7; at the time of discharge, his neck pain and swelling had resolved and he was clinically euthyroid. He was discharged with a tapering course of oral prednisone and oral ibuprofen, to have close follow-up with his primary care physician as well as an endocrinologist.

## Discussion

FUO is defined as a temperature of at least 38.3°C for at least three weeks with an unknown diagnosis after an extensive workup for at least one week [1]. FUO can be caused by viral, bacterial, and fungal infections as well as rheumatic, malignant, and inflammatory conditions [1], and thyroid disease is a rare etiology for FUO [2]. SAT is a relatively uncommon diagnosis with an incidence of 12.1 per 100,000 per year [3]. It may present similar to infectious thyroiditis, but differentiating the two can be challenging; nonetheless, it is prudent as the treatment is completely different. Both infectious thyroiditis and SAT may present as painful thyroiditis, but the pathophysiological process behind the two is similar but different as infectious thyroiditis is usually due to an infectious source whereas SAT is solely due to inflammation [4,5]. 

SAT presents with diffuse anterior neck swelling. It usually has a seasonal pattern in keeping with a peak incidence of viral infections such as echovirus and coxsackievirus [6-8]. Most recently, the coronavirus disease 2019 (COVID-19) infection has been linked to SAT based on case reports [1,9]. Human leukocyte antigen (HLA) B35 has also shown some association with SAT [6,9]. The pathophysiology is attributed to a post-viral infection inflammatory process, evidenced by elevated inflammatory markers such as ESR and CRP [1,4]. It results in follicular cell damage, mediated by T lymphocytes and histiocytes circulating around colloid masses, giving a giant cell appearance [4-6]. In contrast, infectious thyroiditis presents with unilateral anterior neck swelling and occurs as a result of bacterial, mycobacterial, fungal, or parasitic infections, in the presence of predisposing conditions such as congenital abnormalities, immunosuppression, and older age [4]. 

Hyperthyroidism is present in about half of the cases of SAT, due to leakage of preformed thyroid hormones from damaged follicular cells. This results in elevated T4 and T3 and suppressed TSH levels, which are transient and may last up to three to six weeks [8]. The relative hyperthyroid state is followed by a period of hypothyroidism which can last up to six months and infrequently can be permanent, but most patients return to a euthyroid state [4,9]. Anti-thyroid peroxidase and anti-thyroglobulin antibodies are generally low, and thyroglobulin is elevated [1,8].

It is also important to distinguish hyperthyroidism due to Graves' disease and SAT as treatment is different. The distinction can be made based on serology and imaging with a radioactive iodine uptake scan. In SAT, serology usually demonstrates a hyperthyroid state with elevated inflammatory markers and imaging features of low radioactive iodine uptake. Other distinguishing features in favor of SAT include absent or low anti-thyroid peroxidase antibody and anti-thyroglobulin antibody, but thyroglobulin may be elevated. In SAT, ultrasound with Doppler usually reveals gland enlargement with diffuse or focal hypoechoic lesions and hypovascularity and depressed radioactive iodine uptake on radioactive iodine imaging [1,9]. On the other hand, Graves' disease is marked by a positive thyroid-stimulating antibody, hypervascularity of the thyroid gland on Doppler ultrasound, and diffuse enhancement in radioactive iodine uptake [4,8]. 

In our patient's case, he had persistent fevers for approximately one month, not relieved by multiple courses of antibiotic therapy, and though his initial neck soft tissue CT showed features of suppurative thyroiditis, he responded poorly to antibiotic therapy. Thyroid Doppler was done which pointed towards a diagnosis of SAT, for which the mainstay of management is with anti-inflammatory medications such as nonsteroidal anti-inflammatory drugs (NSAIDs) and glucocorticoid therapy [8,10]. NSAIDs can be used in mild and moderate diseases, but prednisone is preferred in severe cases or if there is no response to NSAIDs [10]. Our patient had ongoing symptoms for approximately four weeks and as such was treated with steroids and NSAIDs. There is usually rapid improvement within 48 hours as seen in our patient, who was started on steroid therapy and had defervescence in less than 24 hours [4]. Steroid therapy may be required for four to six weeks but may be needed for longer periods in the setting of prolonged illness. It is important to note that though steroids provide symptom resolution, they do not prevent recurrence nor early or late thyroid dysfunction [4,10]. Unfortunately, up to 10-15% of patients have persistent hypothyroidism, requiring long-term levothyroxine therapy [4,10]. Therefore, outpatient follow-up is recommended for regular monitoring with thyroid function tests, as late-onset hypothyroidism can occur [11]. At the time of discharge, our patient was clinically euthyroid and was given appropriate endocrinology outpatient follow-up. 

## Conclusions

SAT can manifest with clinical features resembling infectious thyroiditis or Graves' disease, potentially leading to the misinterpretation of fevers as of infectious origin. It is crucial to note that SAT does not respond to antibiotic therapy and may present as FUO. Accurate diagnosis relies on a comprehensive approach encompassing a detailed medical history, physical exam making note of painful or painless thyroid, and pertinent investigations such as Doppler ultrasound, radioactive iodine uptake scan, and serologic markers of inflammation. Early recognition facilitates the initiation of prompt and effective treatment, typically involving steroids and/or NSAIDs, leading to favorable outcomes. Unfortunately, some patients may have early or late thyroid dysfunction despite appropriate therapy. In our patient's case, he had a good response to treatment and was discharged with close outpatient follow-up. 
